# Study on Proteomics-Based Aortic Dissection Molecular Markers Using iTRAQ Combined With Label Free Techniques

**DOI:** 10.3389/fphys.2022.862732

**Published:** 2022-07-15

**Authors:** Ting Deng, Yongguang Liu, Akindavyi Gael, Xiaohua Fu, Xiaofang Deng, Yunfeng Liu, Yizhang Wu, Yingzhi Wu, Huimin Wang, Yuying Deng, Jun Lai, Qiang Fu

**Affiliations:** ^1^ Department of Cardiovascular Disease, First Affiliated Hospital of Guangzhou University of Chinese Medicine, Guangzhou, China; ^2^ Department of Cardiology, Laboratory of Heart Center, Heart Center, Zhujiang Hospital, Southern Medical University, Guangzhou, China; ^3^ Guangdong Provincial Biomedical Engineering Technology, Research Center for Cardiovascular Disease, Guangdong, China; ^4^ Sino-Japanese Cooperation Platform for Translational Research in the Heart Failure, Guangzhou, China; ^5^ Department of Organ Transplantation, Zhujiang Hospital, Southern Medical University, Guangzhou, China; ^6^ Department of Cardiology, Shenzhen Hospital, Southern Medical University, Shenzhen, China; ^7^ Department of Invasive Technology, Zhujiang Hospital, Southern Medical University, Guangzhou, China; ^8^ Department of Neonatology, Guangdong Provincial People’s Hospital, Guangzhou, China

**Keywords:** aortic dissection, proteomic, iTRAQ, label-free, Lumican

## Abstract

**Background:** Aortic dissection refers to the separation of aortic media and extension along the long axis to form the true and false chambers of the aortic wall. 65–70% of the patients died of cardiac tamponade, arrhythmia, dissection rupture, etc. At present, echocardiography, computed tomography angiography (CTA), etc. are the main diagnosis tools for aortic dissection. To date, there is no rapid serum molecular marker that can be used for differential diagnosis and risk assessment.

**Objectives:** To screen serum molecular markers systematically amid aortic dissection and acute coronary syndrome and to preliminarily identify the pathogenesis of acute aortic dissection.

**Methods:** Related disputes cases of all hospitals were statistically analyzed for the AAD medical disputes ratio, early death ratio and misdiagnosis ratio from the database of Guangdong Province Medical Disputes Coordination Committee from 2013 to 2017. Serum and Aortic tissues samples were respectively quantified by iTRAQ and label-free analysis, further validated by ELISA and protein verified by immunofluorescence and Western blot from AAD and control patients enrolled from the Zhujiang Hospital of Southern Medical University and Guangdong Province people's Hospital from 2016 to 2018.

**Results:** AAD cases ratio accounted for 15.29% in all 150 cardiovascular disputes, 59.26% in all cardiovascular death less than 24 h, and 88.89% in the patients who remained undiagnosed at the time of death, 84 proteins (66 and 18 upregulated and downregulated, respectively) were identified by iTRAQ and 16 proteins (9 and 7 upregulated and downregulated, respectively) by Label-free. Nine proteins (Lumican, FGL1, PI16, MMP9, FBN1, MMP2, VWF, MMRN1, and PF4) related to the pathogenesis of aortic dissection were identified by David /Ease and String techniques as candidate biomarkers for verification test. Four proteins (Lumican, FGL1, PI16, and MMP9) were found to be statistically different after ELISA verification. The expression of FGL1, PI16, and MMP9 proteins was pathologically significantly increased except for Lumican. Histologically, TGF-β1, α-SMA, and Collagen1 were also significantly higher in the aortic group.

**Conclusion:** Lumican, FGL1, PI16, and MMP9 may be potential biomarkers in AAD patients, and the Lumican-mediated TGF-β1 pathway is likely to be involved in the pathogenesis of aortic dissection.

## 1 Introduction

Acute aortic dissection (AAD) is a major vascular disease with high mortality and poor prognosis. As a life-threatening cardiovascular disease, the related untreated mortality rate is about 1–2% per hour after the onset of symptoms (Hagan et al., 2000). Early diagnosis is crucial and can even save lives (Klompas, 2002). One of the main challenges in establishing the diagnosis is to differentiate AAD from other sudden severe chest pain diseases, especially acute myocardial infarction (AMI) and pulmonary embolism (PE), because patients suffering from these diseases present similar symptoms but require different treatments. Misdiagnosis of AAD often leads to catastrophic bleeding or AAD deterioration, especially when thrombolytic drugs are used improperly (Wilcox et al., 1988; Butler et al., 1990).

According to 2014 ESC guidelines for the diagnosis and treatment of aortic dissection, imaging remained the main method of diagnosis and differential diagnosis (Suzuki et al., 2008). However, CTA, transthoracic or transesophageal ultrasound, and other imaging methods are time-consuming and present various risks in the process of examination. The detection of serological markers is more objective, and blood marker testing can reduce patient movement. Unfortunately, there are currently no widely available AAD biomarkers with high sensitivity and specificity. At present, research on aortic dissection markers are mainly based on the pathophysiological development process of the disease, which mainly includes smooth muscle protein, matrix metalloprotease, elastic protein fragment, D-dimer, and some other proteins. However, these molecules cannot be clinically widely used because of the lack of specificity and sensitivity.

The combination of isobaric Tags for Relative and Absolute Quantitation (iTRAQ) and label-free methods can be used to identify biomarkers of various diseases (Wen et al., 2009). Combining these two methods, we proceeded to determine the serum biomarkers released after aortic dissection, which can provide a wider range of proteomics for AAD diagnosis. Discovery of new molecular markers, the diagnosis of aortic dissection can be more sensitive and specific, reduce the dependence on imaging equipment and physicians’ experience, provide reliable and economical tools for lower-level hospitals that lack the support of relevant conditions, thereby reducing misdiagnosis and delays, and achieve the purpose of optimal diagnosis and treatment.

## 2 Materials and Methods

### 2.1 Ethical Approval of the Study Protocol

**FIGURE 1:**
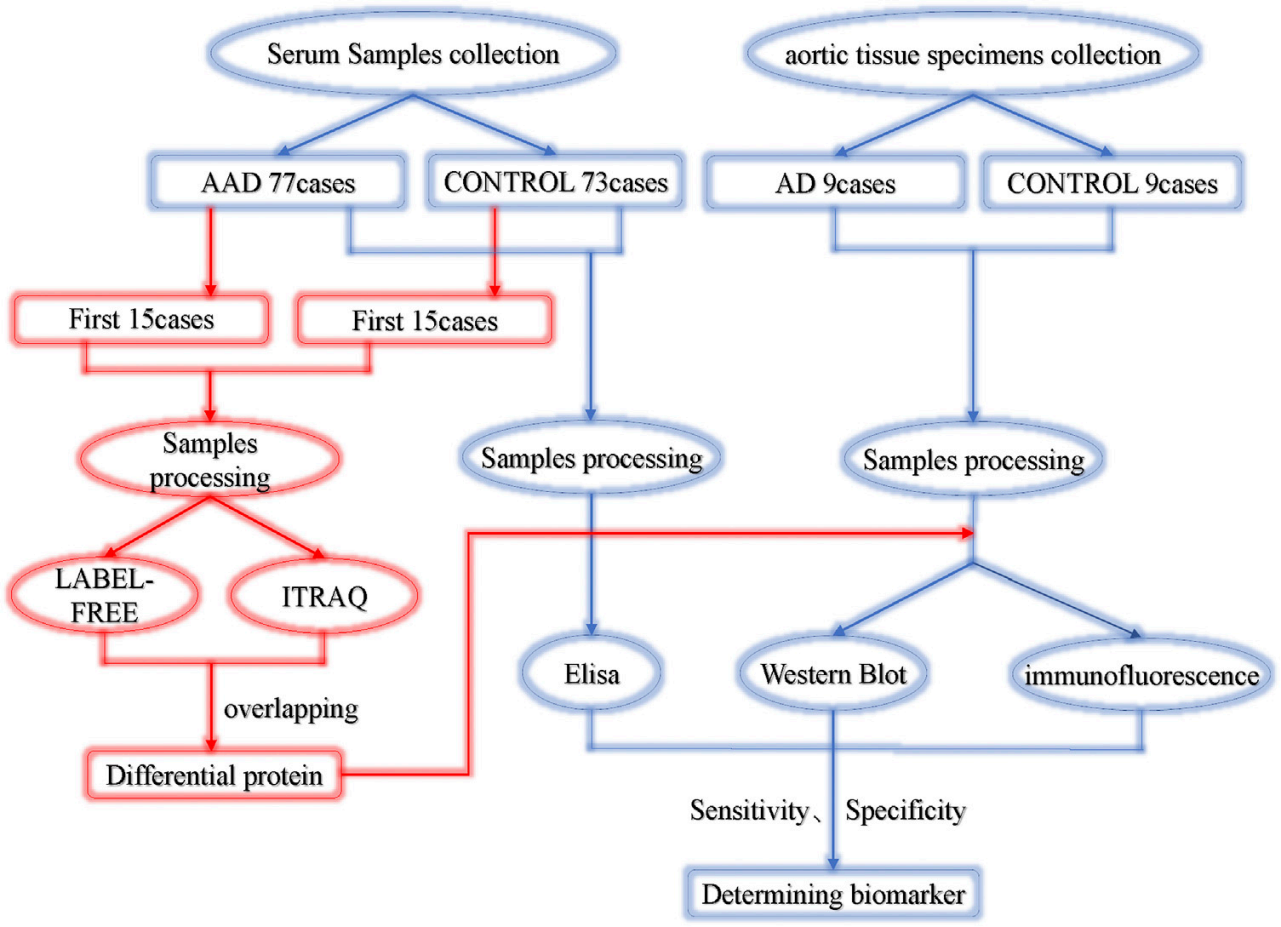
Experimental design. AAD, acute aortic dissection; ACS, acute coronary syndrome.

Written informed consent was obtained from all the participants before enrollment. The protocol of this study was carried out according to the principles of the Declaration of Helsinki and approved by the Medical Ethics Committee in Zhujiang Hospital (Case No: 2018-XXGNK-001). The protocol is available at www.clinicaltrials.gov (ChiCTR1800015743). This trial was conducted and monitored according to the guidelines for Good Clinical Practice. The design of this study is shown in Figure 1.

### 2.2 Analysis of Cardiovascular Disputes Cases

**FIGURE 2:**
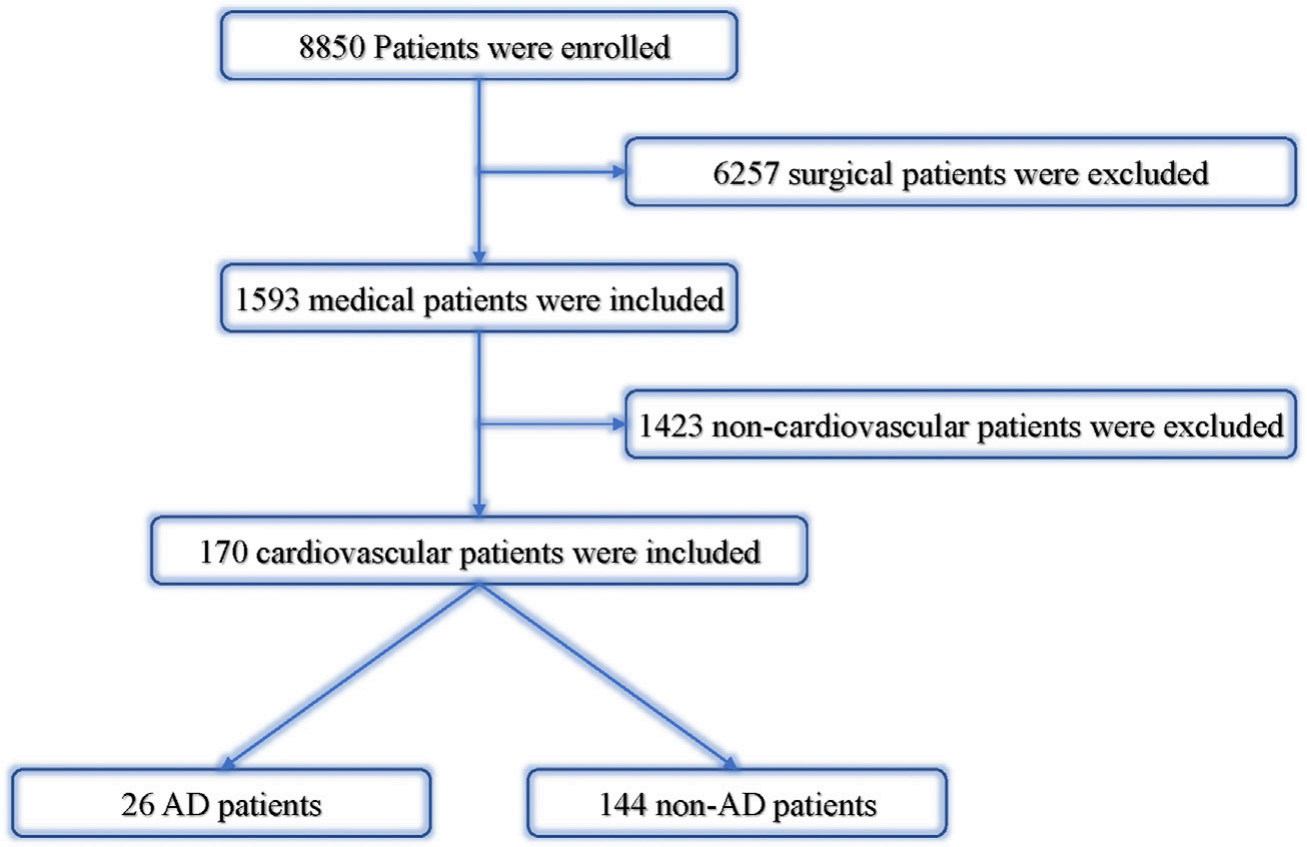
Analysis of cardiovascular dispute cases. AD, aortic dissection; non-AD, without aortic dissection.

We collected cardiovascular dispute cases registered in the database of the Guangdong Province Medical Disputes Coordination Committee in all hospitals in the Guangdong Province from January 2013 to December 2017. Patients were divided into two groups (AAD group and non-AAD group). The overall mortality rate, 24 h mortality rate, and undiagnosed mortality rate were separately calculated according to different levels of hospitals (Figure 2).

### 2.3 Analysis of Clinical Sera and Tissues Samples

#### 2.3.1 Materials Collection

Seventy-seven AAD patients (AAD group) with sudden chest pain within 72 h and 73 patients with non-AAD chest pain were enrolled from the Zhujiang Hospital of Southern Medical University and Guangdong Province people’s Hospital for serum analysis from January 2016 to December 2018. Nine AAD patients and nine organ donors’ aortic tissues were collected from the same institutions. Serum samples were collected immediately after admission and kept at a temperature of 4°C for 1 h and centrifuged at 3,000 rpm for 15 min. After obtaining informed consent, the aortic tissue specimens were retrieved, rinsed with PBS, labeled, aliquoted, and quickly put into liquid nitrogen for rapid freezing. Both serum and tissue specimens were stored at −80°C until further analysis.

##### 2.3.1.1 Serum Collection

The inclusion criteria were as follows: 1) age: ≥18 years old; 2) no gender restrictions; 3) patients with chest pain within 72 h; 4) patients with acute aortic dissection confirmed by aortic CTA or angiography. The exclusion criteria were as follows: 1) pregnant or lactating women; 2) history of cardiopulmonary resuscitation (CPR) and cardiac interventional therapy or surgery in 1 week; 3) severe hepatorenal insufficiency; 4) patients in shock requiring vasoactive drugs on admission. The first 15 patients in the AAD group and control group were analyzed by iTRAQ and label-free. All enrolled patients’ serum was tested for ELISA. The clinical features of the two groups were summarized (Table 1). The average age of AAD and ACS patients was 56.93 ± 13.24 years and 63.87 ± 12.39 years, respectively. ELISA was used to verify the clinical features of 77 AAD and 73 non-AAD patients as summarized (Table 2). The mean age of AAD patients was 57.88 ± 12.33 years. The average age of ACS patients was 59.34 ± 10.12 years. There was no significant difference in age distribution between the two groups (*p* > 0.05).

**TABLE 1:** Clinical features of the iTRAQ, label-free analysis subjects.

	AAD	Control	P value
N	15	15	/
Age (mean±SD)	56.93 ± 13.24	63.87 ± 12.39	0.150^a^
Gender (male/female, n)	14/1	13/2	1.000^b^
Hypertension (N)	14	8	0.035^b^
Smoke (N)	10	6	0.272^b^
Diabetes	1	4	0.330^b^
Weight	73.83 ± 11.16	60.37 ± 8.40	0.001^a^
Height (N)	1.69 ± 0.04	1.68 ± 0.07	0.701^a^
BMI	25.73 ± 3.38	21.3 ± 2.22	<0.001^a^
HGB	137.4 ± 18.5	128.7 ± 15.0	0.171^a^
PLT	196.4 ± 95.1	267.4 ± 94.4	0.050^a^
RBC	4.44 ± 0.70	4.43 ±1.01	0.962^a^
WBC	12.33 ± 5.08	9.15 ± 2.40	0.037^a^
ALT	19.5 (13.3, 27.5)	36.5 (27.0, 59.0)	0.007^C^
AST	19.0 (16.0,32.0)	76.0 (27.8, 218.5)	0.004^C^
CK	95.0 (69.0, 209.0)	177.0 (99.8, 2307.1)	0.081^C^
CKMB	8.7 (6.3, 16.4)	25.6 (15.5, 182.3)	0.001^C^
Creatinine	110.3 (91.7, 163.8)	120.0 (93.0, 146.0)	0.727^c^
INR	1.07 ± 0.07	1.11 ± 0.20	0.449^a^
APTT	37.6 (34.3, 40.0)	37.7 (33.5, 42.3)	0.852^c^
FIB	3.75 (2.01, 6.04)	3.71 (3.29, 4.06)	0.868^c^

a t-test.

b Chi-square test.

C Mann-Whitney U test. BMI, Body Mass Index; HGB, hemoglobin; PLT, platelets; RBC, red blood cell; WBC, white blood cell; ALT, alanine transaminase; AST, aspartate aminotransferase; CK, creatine kinase; CKMB, creatine kinase-MB; INR, International normalized ratio; APTT, activated partial thromboplastin time; FIB, fibrinogen.

**TABLE 2:** Clinical features of the validation analysis subjects.

	**AAD**	**Control**	**P value**
N	77	73	/
Age (mean±SD)	57.88 ± 12.33	59.34 ± 10.12	0.431^a^
Gender (male/female, n)	60/17	58/15	0.819^b^
Hypertension (N)	69	51	0.003^b^
Smoke (N)	48	46	0.620^b^
Diabetes	20	18	0.853^b^
Weight T	68.72 ± 12.36	63.22 ± 10.02	0.003^a^
Height HP (N)	1.66 ± 0.07	1.65 ± 0.08	0.498^a^
BMI	24.99 ± 3.60	23.23 ± 2.90	0.001^a^
HB	132.0 ± 17.2	130.3 ± 30.0	0.670^a^
PLT	208.6 ± 102.2	234.7 ± 85.2	0.092^a^
RBC	4.46 (4.15,4.88)	4.53 (4.09,5.33)	0.319^c^
WBC	11.87 ± 4.29	9.76 ± 3.80	0.002^a^
ALT	19.5 (15.0,29.3)	28.0 (19.5,40.0)	0.006^c^
AST	21.0 (18.0,30.0)	19.0 (26.0,70.0)	0.039^c^
CK	99.9 (65.3,179.5)	139.4 (77.4,666.8)	0.005^c^
CKMB	9.85 (7.93,14.15)	18.85 (12.50,57.05)	<0.001^c^
SCR	9.85 (7.93,14.15)	84.6 (69.0,106.2)	0.092^c^
INR	1.12 (1.05,1.22)	1.03 (0.95,1.12)	<0.001^c^
APTT	39.1 (35.6,42.9)	38.1 (34.7,42.4)	0.543^c^
FIB	4.10 (3.04,6.23)	3.42 (2.95,24.14)	0.024^c^

a t-test.

b Chi-square test.

C Mann-Whitney U test. BMI, Body Mass Index; HGB, hemoglobin; PLT, platelets; RBC, red blood cell; WBC, white blood cell; ALT, alanine transaminase; AST, aspartate aminotransferase; CK, creatine kinase; CKMB, creatine kinase-MB; INR, International normalized ratio; APTT, activated partial thromboplastin time; FIB, fibrinogen.

##### 2.3.1.2 Tissues Samples Collection

The inclusion criteria were as follows: 1) age: ≥18 years old; 2) no gender restrictions; 3) patients with acute aortic dissection confirmed by aortic CTA or angiography; 4) aortic valve replacement is required in patients with AAD ***.*** The exclusion criteria were as follows: 1) pregnant or lactating women; 2) history of CPR and cardiac interventional therapy or surgery in 1 week; 3) severe hepatorenal insufficiency; 4) patients in shock requiring vasoactive drugs on admission; 5) hereditary aortic dissection patients. The average age of AAD patients was 49.3 ± 6.61 years old. The average age of organ donors was 50.1 ± 7.51 years old (Table 3).

**TABLE 3:** Clinical features of Western blot, immunofluorescence analysis subjects.

	**AD**	**CONTROL**	**P**
N	9	9	/
Age (mean±SD)	49.33±6.61	50.11±7.51	0.819^a^
Gender (male/female, n)	8/1	8/1	1.000^b^
Hypertension (N)	7	5	0.620^b^
Smoke (N)	6	5	1.000^b^
Diabetes	0	0	1.000^b^
HB	130.8 ± 19.6	107.6 ± 38.9	0.136^a^
PLT	220.0 (164.5,283.0)	125.0 (82.5,264.0)	0.401^c^
RBC	4.28 ± 0.66	3.53 ± 1.10	0.097^a^
WBC	9.43 (7.57,15.76)	11.30 (6.68,17.70)	0.895^c^
ALT	21.0 (11.5,40.0)	21.0 (18.0,63.5)	0.331^c^
AST	20.0 (18.0,25.5)	53.0 (25.5,126.5)	0.007^c^
CK	95.0 (40.0,136.5)	299.8 (79.5,1862.7)	0.102^c^
CKMB	14.5 (10.5,22.8)	40.1 (19.2,44.5)	0.019^c^
CR	87.6 (68.7,105.5)	146.0 (96.3,210.6)	0.024^c^
DDI	2080 (1275,14695)	7580 (2865,10000)	0.400^c^
INR	1.18 ± 0.12	1.44 ± 0.40	0.095^a^
APTT	46.4 ± 9.0	40.8 ± 12.0	0.279^a^
FIB	4.61 ± 2.04	7.00 ± 2.14	0.027^a^

a t-test.

b Chi-square test.

C Mann-Whitney U test. BMI, Body Mass Index; HGB, hemoglobin; PLT, platelets; RBC, red blood cell; WBC, white blood cell; ALT, alanine transaminase; AST, aspartate aminotransferase; CK, creatine kinase; CKMB, creatine kinase-MB; INR, International normalized ratio; APTT, activated partial thromboplastin time; FIB, fibrinogen.

#### 2.3.2 Biomarker Verification Method

##### 2.3.2.1 iTRAQ Multiplex Labeling and Chromatography

The 14 most abundant proteins were retrieved from each group of serum samples using Agilent multiple affinity removal liquid chromatography column-Human 14 (Agilent Technologies, Santa Clara, United States). Each sample was dissolved, reduced, and alkylated, then digested overnight with trypsin [w (trypsin): w (protein) = 1:20] at 37°C. ITRAQ reagent labeling was carried out in accordance with AB SCIEX’s iTRAQ labeling kit specification (Zhang et al., 2016). ITRAQ 118 119 121 was used in the control group and iTRAQ 113 114 117 was used in the AAD group. The marked peptides of each group were mixed and graded by the Agilent 1260 Infinity II HPLC system (Zhai et al., 2013).

##### 2.3.2.2 Label-Free Sample Preparation and MS Analysis for Experiments

Agilent multiple affinity removal LC column-human 14 of the corresponding samples was used to remove the high abundance protein, and the low abundance component solution was obtained. Mass spectrometry was performed after FASP digestion, and each sample was separated by a nanoliter flow rate Easy nLC system. Buffer A solution was 0.1% formic acid aqueous solution and B solution was 0.1% formic acid acetonitrile aqueous solution (acetonitrile is 80%). The chromatographic column was equilibrated with 100% liquid A, and the samples were separated from the automatic sampler to the analytical column (Thermo scientific, Acclaim PepMap RSLC 50 µm × 15 cm, nano viper, P/N164943) at a flow rate of 300 nl/min. After chromatographic separation was carried out, samples were analyzed by a Q-Exactive Plus spectrometer. The analysis time was 60 min (Zhang et al., 2016).

##### 2.3.2.3 Bioinformatics Analysis

For the analysis of iTRAQ and label-free markers, the ProteinPilot search engine (AB Sciex) was used for protein identification by searching for human species in the UniProtKB/Swiss-Prot database. For the bioinformatics analysis of differentially expressed proteins, GeneGO MetaCoreTM soft (https://portal.genego.com/) was used. After comparing proteins in label-free and iTRAQ, common target-related proteins were found to appear in both lists. In addition, the possible signal network of BF may be predicted by the automatic expansion of common target-related proteins; this emphasizes the most unique approach in the network, including the pathways that include the most protein nodes, it also shows the subcellular location of proteins in the network. Finally, we used GO seq for the GO enrichment analysis, pathway enrichment analysis was assessed by hypergeometric test, protein interaction prediction was confirmed by protein interaction database String, and the Cytoscape software was used for interaction network display.

#### 2.3.3 ELISA Analysis

We used Lumican (abcam, United States), PF4 (abcam, United States), VWF (abcam, United States), MMP9 (R&D Systems Company, United States), MMP2 (R&D Systems Company, United States), MMRN1 (Biomatik, United States), FGL1 (Biomatik, United States), FBN1 (Biomatik, United States), and PI16 (abbexa, United Kingdom) to detect the aforementioned proteins by the enzyme-linked immunosorbent assay, ELISA. The procedure was carried out in strict accordance with the manufacturer’s instructions, the samples were diluted 20–100 times differently, the original standard provided by the kit was gradually diluted, and the standard curve was used to calculate the biomarker concentration in the sample.

#### 2.3.4 Immunofluorescence Staining

The formalin-fixed, paraffin-embedded aortic sections were deparaffinized, dehydrated, antigen-repaired, and blocked with 5% BSA for 40 min at room temperature, followed by PI16 (1:200, NVUS, United States), FGL1 (1:200, NOVUS, United States), MMP9 (1:100, Abcam, United States), and LUM (1:100, Abcam, United States) at 4°C overnight. The sections were washed with PBS and then incubated with a secondary antibody conjugated to Alexa Fluor dye (1:500) for 2 h at room temperature. The nuclei were stained with DIPA for 5 min at room temperature. Images were captured using the Image-Pro Plus V4.5 software. Fluorescence was detected by using a Leica SP5 confocal microscope (Leica Microsystems Inc., Wetzlar, Germany). The magnification was 200 folds and the average signal intensity normalized to the aortic region between the groups was compared.

#### 2.3.5 Western Blot Analysis

Western blot was utilized on aortic tissue samples from nine patients with aortic dissection and nine normal organs donor to verify the results of four proteins (Lumican, FGL1, PI16, and MMP9). Briefly, 20 µg of protein were separated by 10% SDS-PAGE gel and transferred to the nitrocellulose membrane. The membrane was blocked with 5% skim milk overnight, then incubated at 4°C with PI16 (1:500, NOVUS, United States), FGL1 (1:500, NOVUS, United States), MMP9 (1:1,000, Abcam, United States), Lumican (1:1,000, Abcam, United States) overnight, and GAPDH (1:10,000, Bioworld, United States) antibodies and was then incubated with anti-rabbit or anti-mouse secondary antibodies (Boster, China) at a dilution of 1:8,000. Signals were detected using an enhanced chemiluminescence reagent (Pierce). Band density was quantified using the Image-Pro Plus software, and fold changes in Lumican, PI16, MMP9, and FGL1 densities were normalized to positive control and GAPDH levels.

## 3 Statistical Analysis

All statistical analyses were performed using SPSS 20.0 (SPSS, Chicago, USA) and GraphPad Prism 5 (GraphPad Inc. La Jolla, CA). Data are expressed as the mean ± SD and medians (interquartile range or n (%)). All continuous variables were tested for the normal distribution of data with the Shapiro-Wilk test. Comparisons of continuous variables between two groups were conducted with the independent-samples T-test or the Mann-Whitney U test. The chi-square test was performed to compare qualitative parameters between two groups. Independent and joint detection of candidate biomarkers were amalyzed by receiver operator characteristic (ROC) curve and logical regression modeling. *p* < 0.05 was considered statistically significant.

## 4 Results

### 4.1 AAD Distribution Within Cardiovascular Disputes Cases

**FIGURE 3:**
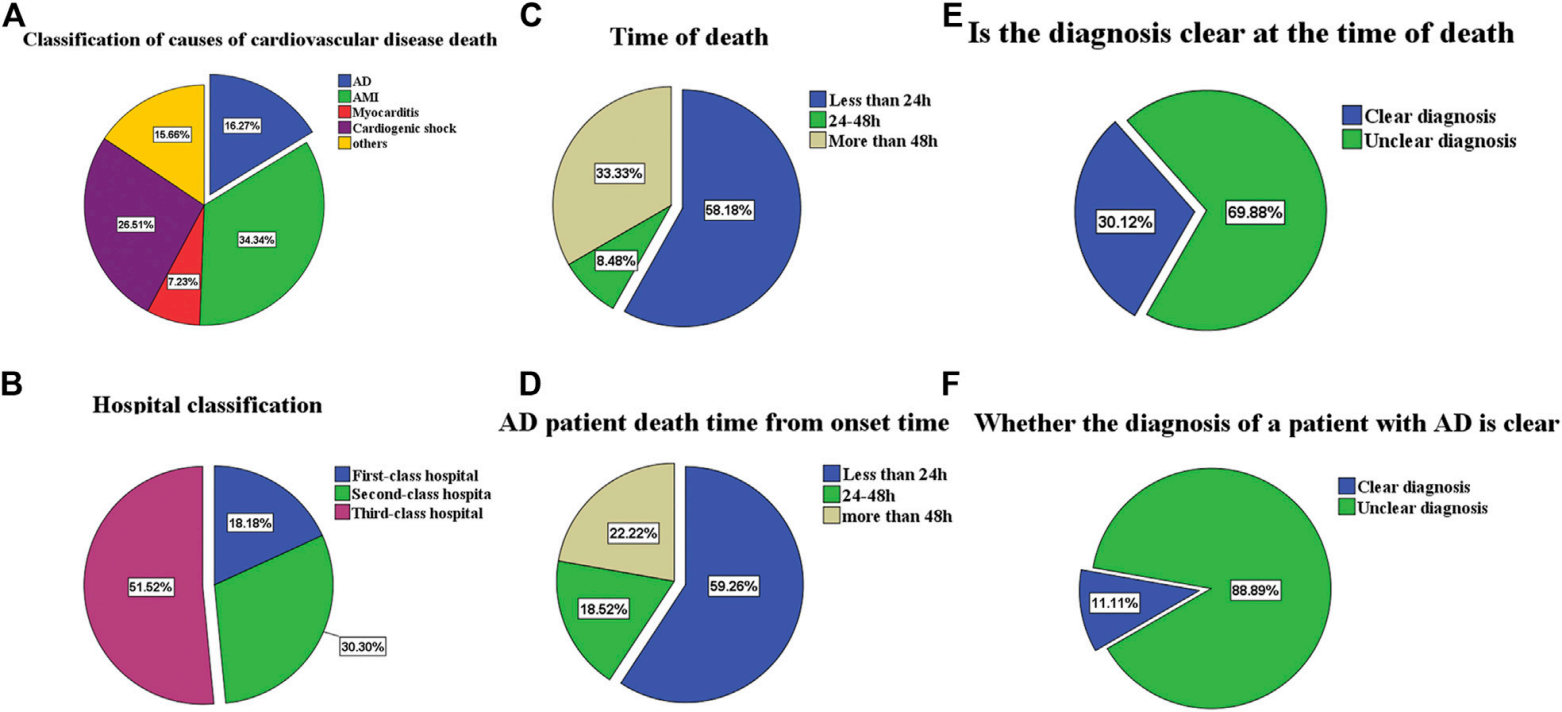
**(A)** is the proportion of deaths from cardiovascular patients. **(B)** is classification of hospitals for the patients. **(C)** is the proportion of different death times of cardiovascular patients. **(D)** is AD patients death time from onset time. **(E)** is the proportion of unexplained death at death. **(F)** is the proportion of unexplained death at death for AD patients.

In the past 5 years, the AAD cases ratio accounted for 16.27% in all cardiovascular disputes, 84.6% in all cardiovascular death less than 48 h, and 88.89% in the patients who remained undiagnosed at the time of death. Overall mortality rate, 24 h mortality rate, and undiagnosed mortality rate were separately calculated according to different levels of hospitals (Table 4; Figure 3).

**TABLE 4:** Clinical features of the medical dispute analysis subjects.

	**AD**	**nonAD**	**P**
N	26	144	/
Age (mean±SD)	47.31 ± 13.55	55.65 ± 16.35	0.015^a^
Gender (male/female, n)	20/6	103/41	0.571^b^
Rate of mortality (diagnosis is not clear)	88.89%	66.40%	0.025^b^
Mortality ratio	—	—	0.784^b^
First-class hospital	5	25	—
Second-class hospital	9	42	—
Third-class hospital	12	77	—

a t-test.

b Chi-square test. AD,Aortic dissection

### 4.2 Identification and Functional Classification of Serum Proteome

**FIGURE 4:**
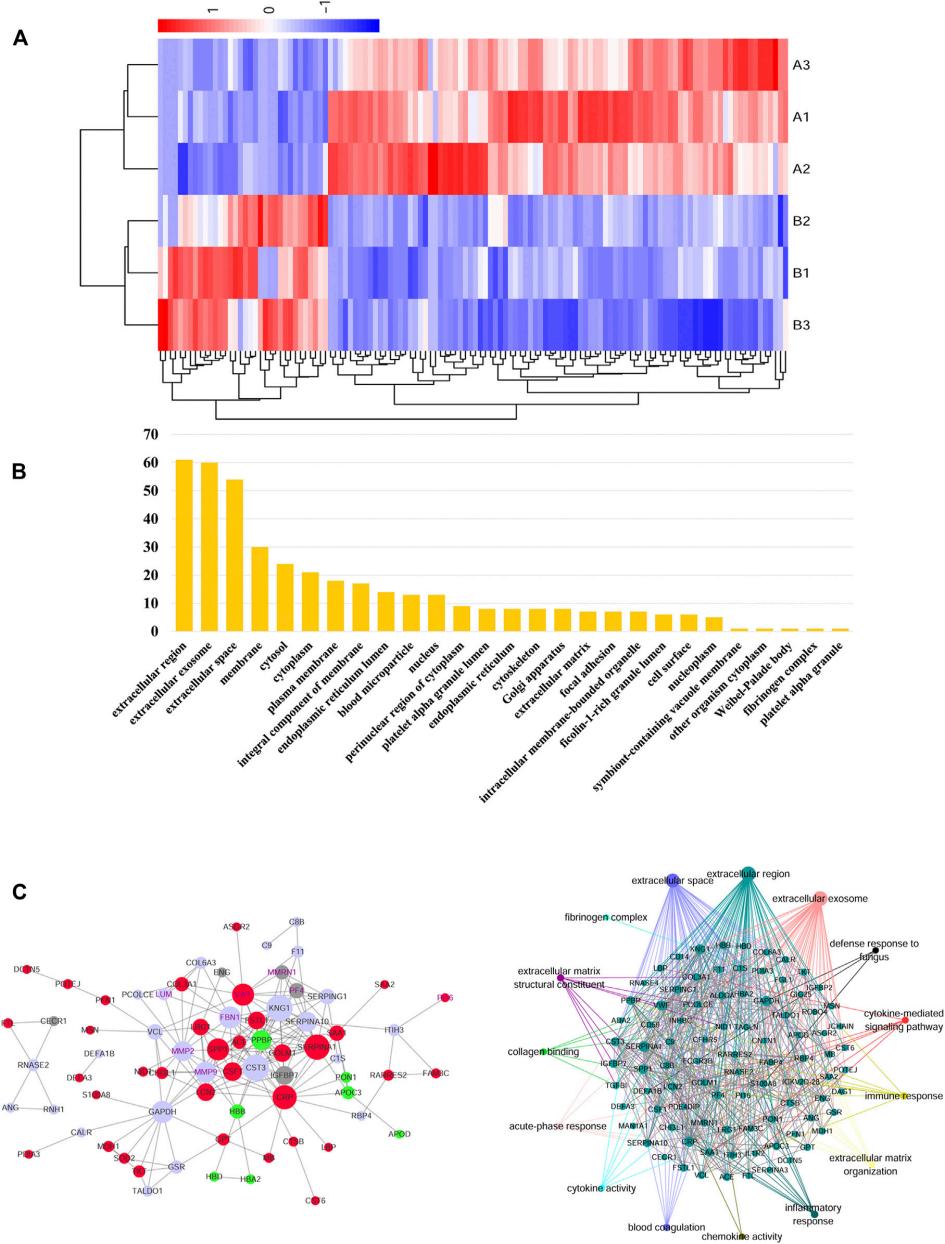
**(A)** In the heat map of the 127 differential metabolites in AAD and ACS, the colors changing from blue to red indicate more metabolites. In the correlation network of metabolites with |*r*| > 1, red plots indicate upregulated metabolites and blue plots indicate downregulated metabolites in the two groups. **(B)** Bioinformatics analysis for the differentially expressed proteins in the AAD and ACS group. Diagram showing the biological process (BP) and cellular component (CC) of differentially expressed proteins of the two groups using the DAVID analysis. **(C)** Network of AAD VS. ACS clustered by the Ingenuity Pathway Analysis. Protein–protein interaction was generated by Cytoscape between the AAD vs. ACS group.

A total of 725 proteins were identified by iTRAQ and 84 were differentially expressed proteins, of which 66 were up-regulated (A/B > 1.2) and 18 were down-regulated (A/B < 1.2). Label-free identified 490 proteins of which 16 were differentially expressed. 9 were up-regulated (A/B > 1.2) and 7 were down-regulated (A/B < 1.2). The cluster analysis of differentially expressed proteins showed that the protein expression in the AAD group was significantly higher than that in the control group (Figure 4A).

According to Gene Ontology (GO), the differential proteins and temptation-free proteins obtained by the two methods were classified according to “molecular function,” “biological process,” and “cell composition,” and sorted according to the number of proteins. The GO analysis showed that the differential protein of the AAD group was concentrated in the extracellular matrix compared with the control group, and the extracellular component was the main component (Figure 4B).

For the biological function analysis, the map was drawn based on the proteins with the highest scores in the IPA and PPI analysis. Exploring the global PPI that may use Cytoscape, we found that FGL1, PI16, MMP9, and Lumican were involved in platelet aggregation, blood coagulation, and many other processes. At the same time, FGL1 improved TGF-β1-induced fibrosis by regulating hemostasis, platelet aggregation, coagulation, and many other processes (Figure 4C).

For screening biomarkers, the study selected upregulated secretory proteins (proteins that could not be downregulated by ELISA). Considering that the most abundant protein albumin in the serum may be affected by liver function, infection, nutrition, and many other factors, it was excluded from the verification list. Therefore, four proteins were provided for verification: FGL1, PI16, MMP9, and Lumican.

#### 4.3.1 Verification of Candidate Molecular Markers

**FIGURE 5:**
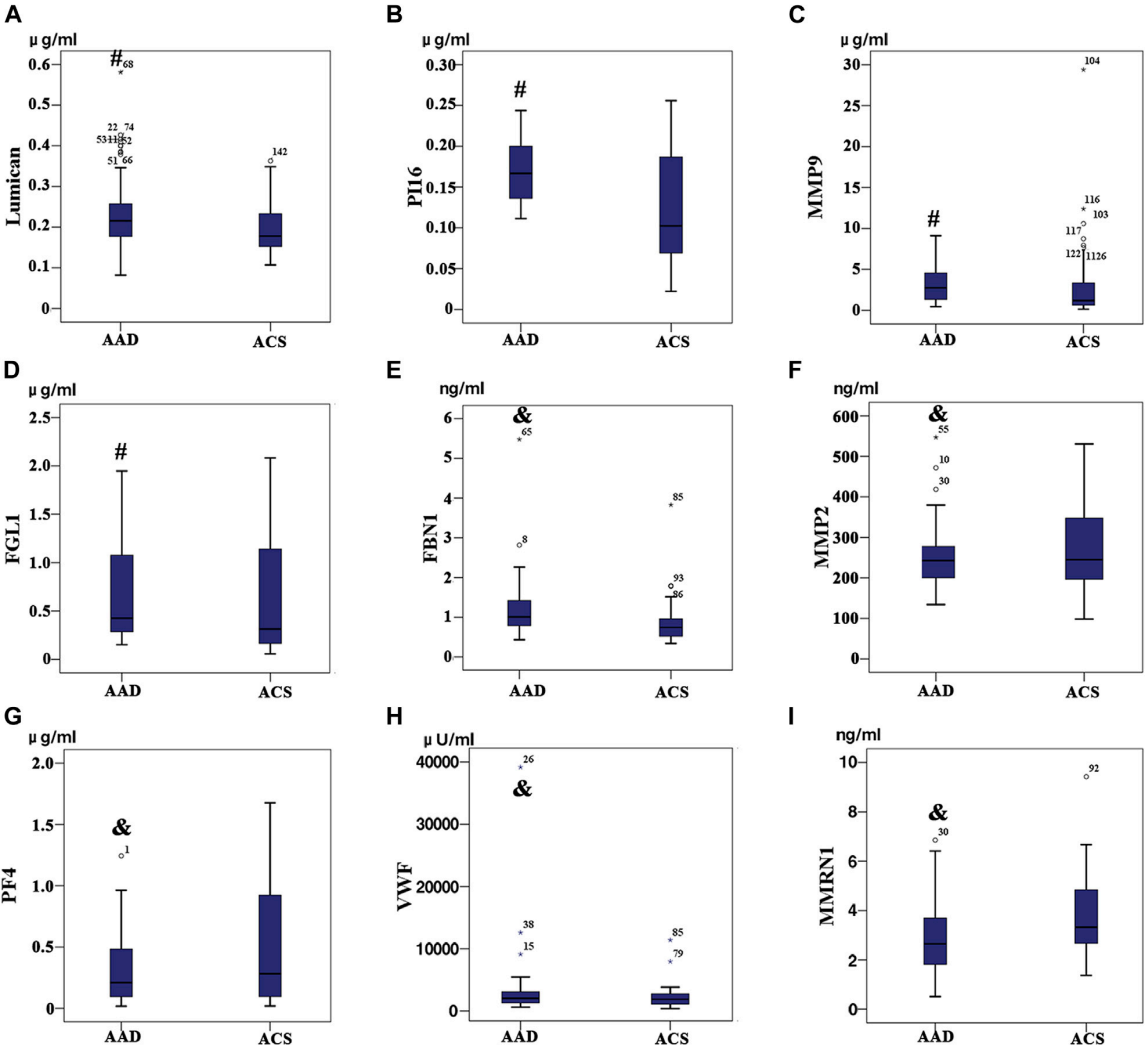
Among the patients with acute aortic dissection (AAD) and Acute coronary syndrome (ACS), **(A)** Lumican, **(B)** Peptidase inhibitor 16, **(C)** Fibrinogen-like protein 1 and **(D)** Matrix metalloproteinase-9 expression level exhibit significant differences between the two group (*p* < 0.05); **(E)** Profibrin 1 antigen (FBN1), **(F)** platelet factor 4 (PF4), **(G)** the Von Willebrand factor (VWF), **(H)** matrix metalloproteinase-2 (MMP-2), **(I)** MMRN1 (*p* > 0.05) expression level did not exhibit any significant differences between the two groups (*p* > 0.05).

Based on iTRAQ and label-free proteomics findings, Lumican, MMP9, PI16, FGL1, FBN1, PF4, MMP2, VWF, and MMRN1 were selected as potential biomarkers for verification targets. Different expressions of individual proteins in AAD (*N* = 77) and non-AAD (*N* = 73) groups (Tables 5, 6) were analyzed. Excluding the proteins with no significant difference between the two groups, four proteins (Lumican, PI16, FGL1, and MMP9) with statistical difference were selected as the final molecular markers. Their expression in aortic dissection group was significantly higher than that in the control group (*p* < 0.05) (Figure 5).

**TABLE 5:** Subset of differentially expressed proteins between the AAD and control groups.

Accession	Name	Biological process	Protein class	AAD:CON	Up/down
P02741	CRP	Acute-phase response	—	11.38	Up
P05109	S100A8	DNA replication	Signaling molecule	3.65	Up
D3DQX7	SAA1	Cellular component movement	Transporter	35.08	Up
Q08830	FGL1	Cell adhesion	Signaling molecule	2.26	Up
P0DJI8	SAA1	Cellular component movement	Defense protein	2.003	Up
Q6UXB8	PI16	—	Immunity protein	1.223	Up
P0DJI8	SAA1	Cellular component movement	Transporter	2.19	Up
P04275	VWF	Cell adhesion	Protease inhibitor	1.536	Up
P14780	MMP9	—	Metalloprotease	1.518	Up
P35555	FBN1	Anatomical structure morphogenesis	Cell adhesion molecule	1.415	Up
P08253	MMP2	—	Metalloprotease	1.099	Down
Q14126	DSG2	Cellular process	Cadherin	1.092	Down
P51884	LUM	Cell growth	—	1.038	Down
O75882	ATRN	Cellular process	Extracellular matrix	1.033	Down
P23142	FBLN1	—	—	0.999	Down

This table lists the highest Unused ProtScores from the upregulated and downregulated proteins. AAD, acute aortic dissection; CON, normal controls.

**TABLE 6:** Label-free with or without protein results.

Protein IDs	Protein IDs	Biological process	Protein class	AAD/Control
P02776	PF4	—	Chemokine	Control
Q5T9B9	ENG	—	—	Control
A0A024RDA6	IGFBP7	Regulation of cell growth	—	AAD
P07737	PFN1	Cellular process	—	AAD
Q08830	FGL1	Cell adhesion	Signaling molecule	AAD
Q13201	MMRN	—	Extracellular matrix glycoprotein	AAD

This table lists all or nothing from the upregulated proteins and downregulated proteins. AAD, acute aortic dissection.

#### 4.3.2 ROC Curve Analysis of the Diagnostic Value of Lumican, PI16, MMP9, and FGL1

**FIGURE 6:**
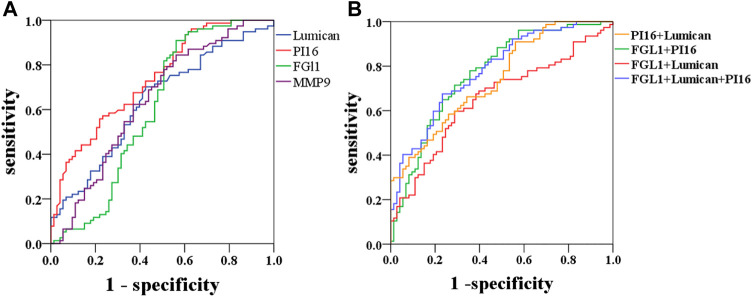
Diagnostic outcomes in the discovery phase are shown via the receiver operating characteristic curve (ROC) curves for PI16, MMP9, and FGL1 to discriminate AAD from controls, *p* < 0.05. PI16 indicates peptidase inhibitor 16, FGL1 indicates fibrinogen-like protein 1, and MMP9 indicates matrix metalloproteinase-9.

The ROC curve was selected for AAD diagnosis by Lumican, MMP9, PI16, and FGL1, and the cutoff value of the maximum Youden index was selected to obtain appropriate sensitivity and specificity. Lumican AUC (0.636), sensitivity 70.1%, specificity 57.5%; PI16 AUC (0.739), sensitivity 55.8%, specificity 78.1%; FGL1 AUC (0.607), sensitivity 90.9%, specificity 43.8%. The AUC (0.641) of MMP9 had a sensitivity of 84.4% and a specificity of 43.8%. PI16 + FGL1 AUC (0.769), sensitivity of 71.4%, specificity of 71.2%; Lumican + PI16 AUC (0.742), sensitivity 90.9%, specificity 43.8%; Lumican + FGL1 AUC (0.653), sensitivity 59.7%, specificity 71.2%. PI16 + FGL1 + Lumican combined with AUC (0.780), sensitivity 67.5%, specificity 76.7% (Table 7; Figure 6). Among all these results, Lumican + PI16 displayed the highest sensitivity and the combination of 3 proteins displayed the highest specificity.

**TABLE 7:** The diagnostic efficiency analysis of four AAD biomarkers.

	AUC	*p*-value	95% CI	Sensitivity (%)	Specificity (%)	Cutoff (ng/dl)	Youden index
LUMICAN	0.636	0.004	0.547–0.725	70.1	57.5	1.865	0.276
PI16	0.739	0.000	0.661–0.816	55.8	78.1	1.973	0.339
FGL1	0.607	0.024	0.512–0.702	90.9	43.8	0.223	0.347
MMP9	0.641	0.003	0.552–0.731	84.4	43.8	0.99	0.282
PI16 + LUM	0.742	0.000	0.665–0.819	90.9	43.8	0.33	0.347
PI16 + FGL1	0.769	0.000	0.693–0.845	71.4	71.2	0.515	0.426
LUMICAN + FGL1	0.653	0.001	0.565–0.741	59.7	71.2	0.517	0.309
LUMICAN + FGL1 + PI16	0.780	0.000	0.707–0.852	67.5	76.7	0.675	0.442

Logical regression analysis of biomarkers in the diagnosis of AAD: the combined prediction model was established using Lumican test results and PI16 and FGL1 as independent variables and dependent variables: logarithm (*p*)= ‒4.861+ 0.561 * Lumican + 1.783 * PI16+0.983 * FGL1. Then variables and statistics were substituted into the model (Table 8).

**TABLE 8:** Logistic regression analysis results of AAD diagnosis with Lumican, PI16, and FGL1.

	B	SE	WALD	*p*	OR	95% CI
LUMICAN	0.561	0.278	4.062	0.044	1.752	1.016–3.022
PI16	1.783	0.37	23.189	0.000	5.945	2.878–12.28
FGL1	0.983	0.397	6.126	0.013	2.673	1.227–5.824
CONSANT	−4.861	0.975	24.848	0.000	0.008	

### 4.4 Identification of TGF-β1-Mediated Signaling Pathway Relative Proteins in Aortic Tissues Samples

**FIGURE 7:**
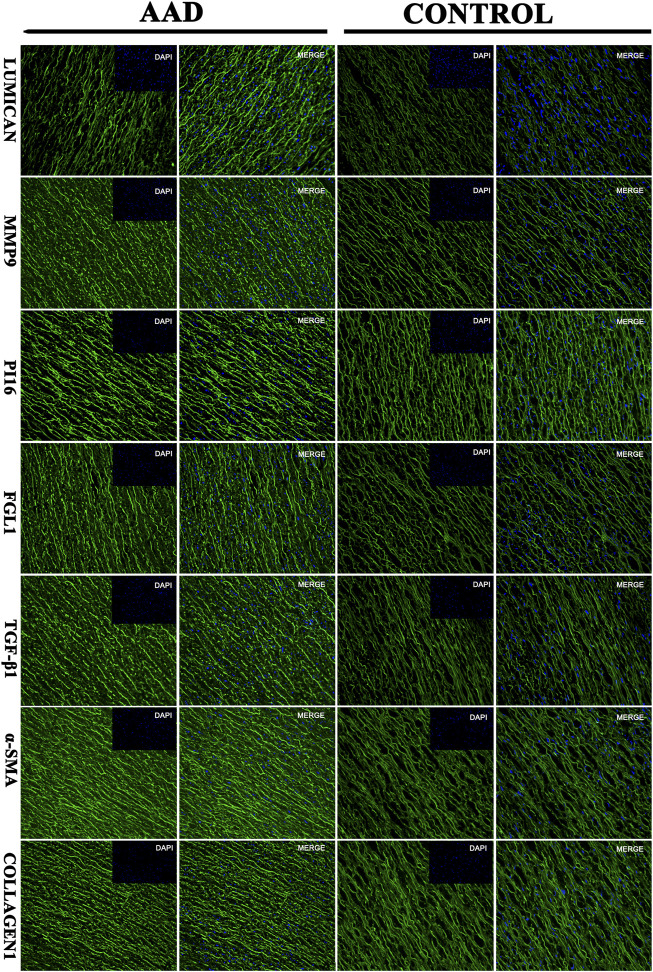
Immunofluorescence: the target protein of the aortic dissection group and the control group (Lumican, matrix metalloproteinase-9 (MMP9), peptidase inhibitor 16 (PI16, peptidase inhibitor 16), fibrinogen-like protein 1 (FGL1, fibrinogen-like protein 1).

**FIGURE 8:**
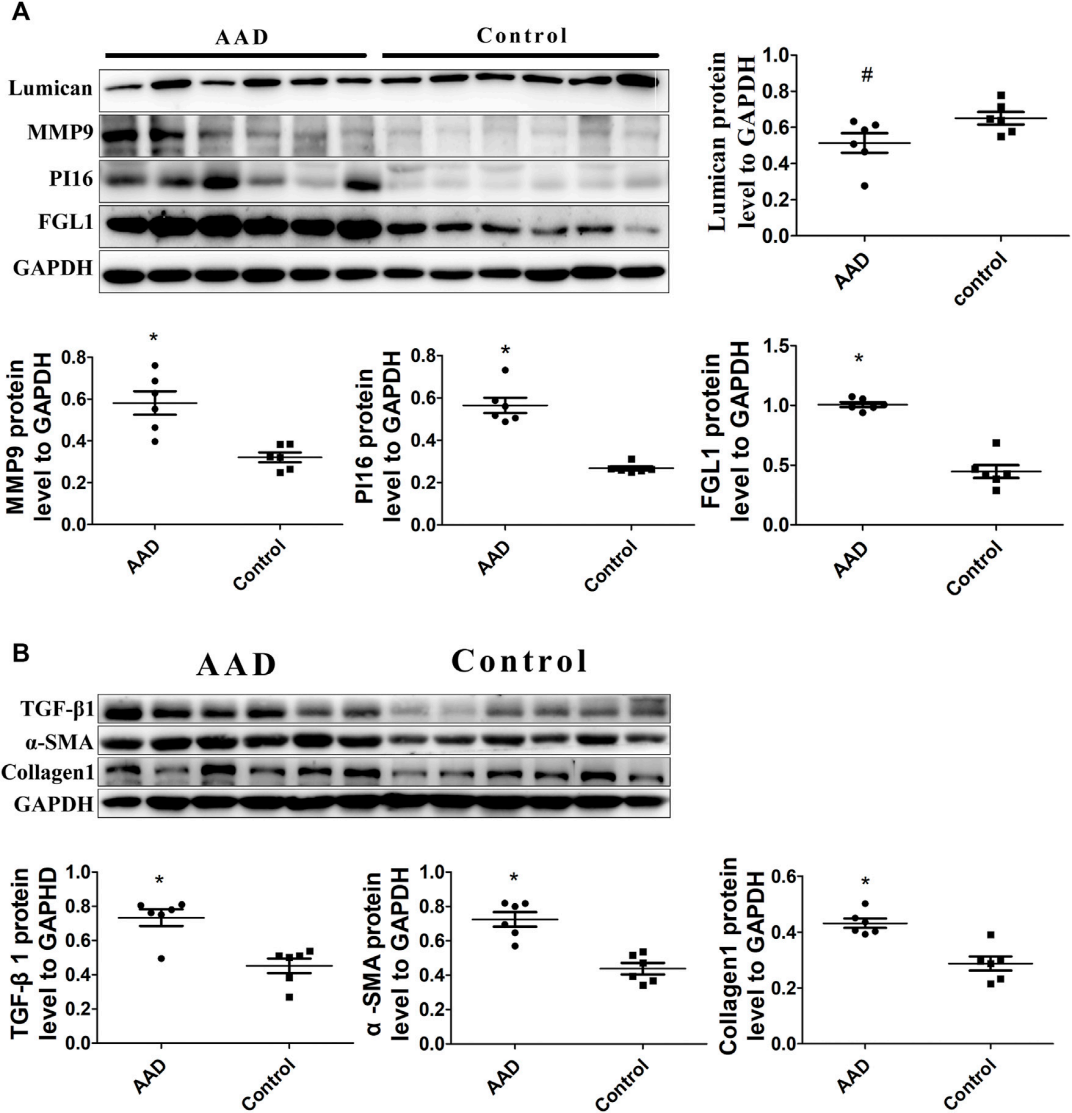
**(A)** Western blot analysis showed increased levels of PI16, FGL1, and MMP9 in aortic dissection (*p* < 0.001), but Lumican is decreased (*p* > 0.05). **(B)** Western blot analysis also showed increased levels of TGF-β1, α-sma, and collagen1 in aortic dissection (*p* < 0.001).

In order to verify the relative proteins involved in the TGF-β1-mediated signaling pathway, immunofluorescence staining and Western blot analyses were performed on the ascending aortic artery samples from the deceased AAD patients and organ donors groups. Fibrosis-related α-sma and collagen 1 were significantly higher in the AAD patients group. PI16, MMP9, FGL1, and TGF-β1 were significantly higher in the aortic wall of AAD patients (*p* < 0.001). Lumican was significantly higher in the serum of AAD patients but no significant difference in the Western blot of human tissue specimens (Figures 7, 8).

## 5 Discussion

AAD is a life-threatening emergency cardiovascular event with a 1–2% increase in mortality every hour after onset. The AAD misdiagnosis rate in the emergency room accounts for 38% (Song et al., 2013). Delayed diagnosis significantly increases the risk of death. Many patients die of aortic rupture or serious complications before a definite diagnosis is established, and a considerable proportion of cases of undiagnosed eventually end up in medical disputes.

Over the past few decades, the number of cases involving medical disputes in some developed countries has increased dramatically, such as the United Kingdom (Goldenberg et al., 2012), the United States (Quraishi et al., 2012), Australia (Jena et al., 2011), New Zealand (Blake, 2010), and Japan (Hiyama et al., 2009). According to a national survey in China, there were 9,831 disputes with 5,519 injured medical personnel in 2006. In 2007, 73.3% of mainland Chinese hospitals reported violent incidents. Because of the sudden occurrence and rapid development of some diseases, some studies have found that patients who die within 48 h are more prone to class I diagnostic errors. A report by the American Institute of Medical Research found that diagnostic errors resulted in about 10% of patients death and as many as 17% of hospital adverse events (McCarthy, 2015). Some studies also found cardiovascular disease were the most common diagnosis in emergency department, and aortic dissection and myocardial infarction caused more death and missed grade I diagnosis (80% and 66.7% per group) (Liu et al., 2017). In this study, we used data from the Guangdong Medical Coordination Committee during 2013–2017. Over the past 5 years, there were a total of 170 cases of medical disputes in cardiovascular patients. Whether different hospitals’ level or physicians’ experience, the AAD medical disputes ratio, early death ratio, and misdiagnosis ratio remain high. The delay in the diagnosis of AAD is a recognized problem (patient transfer risks, imaging time-consuming, and physicians’ misunderstanding) that needs to be improved because the mortality rate is as high as 1%/h before diagnosis (Harris et al., 2011). Contributing to the diagnosis of diseases and benefit patients and caregivers, it is urgent to identify fast, noninvasive, economical, and effective biomarkers.

Cardiac biomarkers, such as for myocardial necrosis (cardiac troponin) and heart failure (natriuretic peptide), have been shown to be successful (Nienaber and Eagle, 2003; Hochholzer et al., 2010; van Kimmenade and Januzzi, 2011). Cardiac troponin is a single biomarker with sufficient sensitivity and specificity, in addition to its favorable release time process, it covers the time window necessary for unambiguity in the clinical environment. According to guidelines, cardiac troponin (cTnT) can furthermore classify ACS and is used as prognosis for MI outcome. Based on a long history of understanding MI mechanism, cTnT has been gradually selected as biomarker for myocardial injury (Cullen et al., 2014). Moreover, current studies are mainly trying to investigate hotspots for AAD markers focused on the pathophysiological development of the disease. Protein markers studies mainly include smooth muscle protein, matrix metalloproteinases, elastin fragments, D-dimer, sST2, and some other proteins, relatively due to various courses of the disease, such as aortic wall degradation, vascular smooth muscle injury, cell necrosis, inflammation, coagulation activation, and extracellular matrix remodeling (Shao et al., 2014). Soluble ST2 has been validated as a biomarker for acute aortic dissection (Wang et al., 2018). The changes of these serum proteins in aortic dissection have been relatively clearly studied, but none of them have good clinical diagnostic value. There is still a long way to determine for AAD pathophysiological mechanism and its biomarkers study. Furthermore discovery and screening researches are still of great value.

Furthermore, with the continuous development and innovation of related technologies proteomics have been clinically used as a marker finding for a long time in the absence of mechanism key points (Arab et al., 2006; Pontillo et al., 2015; de Franciscis et al., 2016; Mesaros and Blair, 2016). Lumican had been reported as a diagnostic marker with sensitivity and specificity of 73.33% and 98.33%, respectively, while it was, respectively, 93.33% and 68.33% for D-dimer (Xiao et al., 2016). Moreover, fibrillin-1, emilin-1, decorin, protein DJ-1, and histone H4 were confirmed as diagnostic markers via Western blot signs of the expression of transforming growth factor-β1 (TGF-β1) signal increase and damaged aortic wall remodeling (Zhang et al., 2015). Withal, fibronectin, and Lumican were selected as differential proteins between the aortic dissection and control group (Gu et al., 2011) and it was found that the expression of extracellular superoxide dismutase SOD was lower than that of the control group (*p* < 0.001) (Liao et al., 2008). In principle, more beneficial quantification with combined high-throughput genomics or proteomics biomolecular data sets methods can acquire greater proteomic coverage than traditional research methods (Kell and Oliver, 2004; Garland et al., 2012; Feala et al., 2013). In this study, iTRAQ and label-free techniques were used to identify serum samples from AAD and ACS patients. The most relevant proteins were selected after a wide range of two overlaps. In this study, a total of nine proteins were found to most likely be tested for serum ELISA in a large number of patients. Three related markers: PI16, Lumican and FGL1 were found, and the ROC curve was drawn to reflect the sensitivity and specificity. The area under the PI16 curve alone was 0.739, the sensitivity was 55.8%, and the specificity was 78.1%, while the area under the PI16 combined with Lumican ROC curve was 0.742, the sensitivity was 90.9%, and the specificity was 43.8%. The area under the ROC curve of the three proteins was 0.78, the sensitivity was 67.5%, and the specificity was 76.7%. The optimal sensitivity and specificity of PI16, Lumican and FGL1 could improve the diagnostic accuracy of AAD. At present, our main diagnosis method still relies on CTA, but CTA takes a long time [18]. Misdiagnosis and delayed diagnosis will lead to medical disputes, and therefore, other simple and inexpensive biomarkers for ADD diagnosis is important in clinical practice.

Based on KEGG (https://www.genome.jp/kegg/pathway.html), Lumican is an important ECM component of the aortic wall that is synthesized in the smooth muscle cells (Qin et al., 2001) of the aortic wall (Naito, 2005). It supports the differentiation of aortic smooth muscle cells and related elastic fiber arrangement and growth, and plays an important role in cell proliferation, migration, and tissue repair (Saika et al., 2003). Jin et al. (2019) found that the knockdown of FGL1 inhibited the production of TGF-β1. In response to profibrotic TGF-β1 signaling, Lumican enhances and accelerates the formation of collagen fibrils, so Lumican expression is positively correlated with profibrotic cytokines (Krishnan et al., 2012). Lumican and FGL1 may be involved in the TGF-β1 signaling pathway-related protein synthesis. The TGF-β1 signaling pathway inducing fibrosis synthesis may play a key role in aortic wall injury and repair. In general, TGF-β1 induced fibrosis participates widely in AAD mechanism such as endothelial malfunction, intima weakening, vascular wall injury and repair, therefore we came up with a hypothesis: the pathogenesis of aortic dissection maybe related to the Lumican-mediated TGF-β1 pathway. Based on immunofluorescence and Western blot of aortic wall specimen of the AAD and organ donors groups, fibrosis-related α-sma and collagen 1 were significantly higher. PI16, MMP9, FGL1, and TGF-β1 were significantly higher in the aortic wall of AAD patients than those of organ donors. These results prove that TGF-β1 mediated fibrosis plays key role in AAD injury and repair process. As for Lumican, previous study showed that Lumican levels was higher in AAD patients than healthy volunteers [42]. Lumican expression was detected in the intima and media of the ascending aorta in patients with AAD [43]. From a clinical point of view, we speculated that the reason for the different expression trends of Lumican in serum and tissue is that Lumican was produced and released into the blood when the aortic dissection tissue re-ruptured, and therefore this protein was highly expressed in the blood compared to that in the tissue. Hence, a hypothesis was put forward: Lumican can be used as an early diagnostic marker for aortic dissection. Lumican activation maybe initially involved as a trigger to the TGF-β1-mediated fibrosis pathway, but further studies still need to be verified through an animal model.

As mentioned above, aortic dissection is a high-risk disease with extremely high mortality. The current pathogenesis is unclear and lacks diagnostic markers of high-specificity and sensitivity. In this study, four effective diagnostic markers were found by combined proteomics methods. Through Elisa verification of a large number of clinical specimens and western-blot verification of aortic tissue specimens, we concluded that: Lumican, PI16, MMP9, and FGL1 protein may be a potential biomarker in patients with aortic dissection, and the pathogenesis of aortic dissection may be related to lumican mediated TGF-β1 pathway. However, there are still some limitations in our study. As a single-center study, our findings should be validated by a multicenter study with a larger population to confirm the diagnostic efficacy and accuracy of this novel assay. In particular, our validation population should include normal people without disease. Future validation studies evaluating Lumican, FGL1, and PI16, and comparing them with other biomarkers, such as NT-proBNP, cardiac troponins and DDI, are thus needed. Finally, further medical image-based confirmatory diagnoses are still essential in clinical practice.

## Data Availability

The datasets presented in this study can be found in online repositories. The name of the repository and accession number can be found below: ProteomeXchange, http://www.proteomexchange.org/, PXD032857.

## Supplementary Material

Supplementary Figure S1 Nomogram for predicting AAD probability.
