# Supplementary figures and images for: Study on Proteomics-Based Aortic Dissection Molecular Markers Using iTRAQ Combined With Label Free Techniques
Source: Front Physiol. 2022 Jul 15;13:862732. doi: 10.3389/fphys.2022.862732 (PMC9335284; doi:10.3389/fphys.2022.862732)

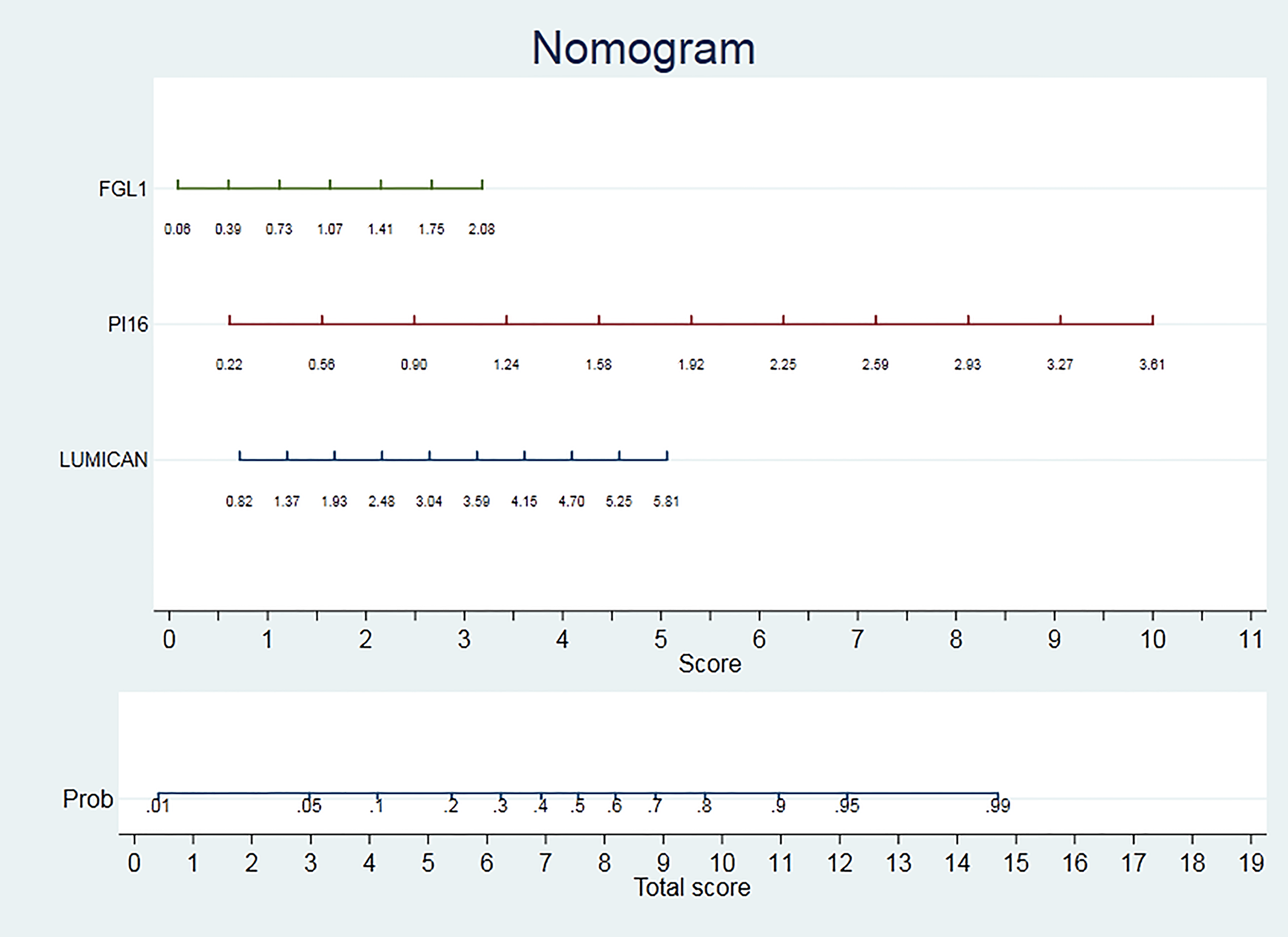

Supplement: Supplementary file 1 [file Image1.tif]
